# The Severity of Nocturnal Hypoxia but Not Abdominal Adiposity Is Associated with Insulin Resistance in Non-Obese Men with Sleep Apnea

**DOI:** 10.1371/journal.pone.0071000

**Published:** 2013-08-12

**Authors:** Anne-Laure Borel, Denis Monneret, Renaud Tamisier, Jean-Philippe Baguet, Patrice Faure, Patrick Levy, Serge Halimi, Jean-Louis Pépin

**Affiliations:** 1 Endocrinology Department, University Hospital, Grenoble, France; 2 Institut National de la Santé et de la Recherche Médicale-INSERM U1042, Grenoble, France; 3 HP2 laboratory, University Grenoble Alpes, Grenoble, France; 4 Department of Metabolic Biochemistry, La Pitié-Salpêtrière Hospital, Paris, France; 5 Sleep, Exercise and Physiology Department, University Hospital, Grenoble, France; 6 Cardiology Department, University Hospital, Grenoble, France; 7 Biology Department, University Hospital, Grenoble, France; University of Tor Vergata, Italy

## Abstract

**Background:**

Beyond obesity, sleep apnea syndrome is frequently associated with excess abdominal adiposity that could contribute to the deteriorated cardiometabolic risk profile of apneic patients.

**Methods:**

The present study addressed the respective contribution of the severity of sleep apnea syndrome and excess abdominal adiposity to the cardiometabolic risk profile of 38 non obese men with polysomnography-diagnosed sleep apnea syndrome (apnea-hypopnea index >15 events/hour). These otherwise healthy men performed a 75g-oral glucose tolerance test (OGTT) with plasma lipid/inflammatory and redox profiles. Twenty-one apneic men with high-waist circumference (>94 cm) were compared to 17 apneic men with low-waist circumference.

**Results:**

Apneic men with high-waist circumference had higher AUC *glucose* and AUC *insulin* than apneic men with low-waist circumference. Accordingly, apneic men with high-waist circumference had higher hepatic insulin resistance as reflected by higher HOMA-resistance index, and lower global insulin sensitivity as reflected by lower insulin sensitivity index of Matsuda (derived from OGTT). The sleep structure and the apnea-hypopnea index were not different between the two groups. However, apneic men with high-waist circumference presented with lower mean nocturnal oxyhemoglobin (SpO_2_). In the 38 men, waist circumference and mean nocturnal SpO_2_ were inversely correlated (r = −0.43, p = 0.011) and were both associated with plasma glucose/insulin homeostasis indices: the higher the waist circumference, the lower the mean nocturnal SpO_2_, the lower the insulin-sensitivity. Finally, in multivariable regression model, mean nocturnal SpO_2_ and not waist circumference was associated with insulin-resistance.

**Conclusion:**

Thus, excess abdominal adiposity in non obese apneic men was associated with a deteriorated insulin-sensitivity that could be driven by a more severe nocturnal hypoxemia.

## Introduction

Obstructive sleep apnea syndrome (OSA) is characterized by recurrent episodes of complete and partial upper airway collapses (episodes of apnea and hypopnea) leading to chronic intermittent hypoxia and ended by micro-arousals. Both the repetition of the desaturation-reoxygenation sequence and micro-arousals are associated with acute surges in sympathetic activity and secondary induce endothelial dysfunction, low grade systemic inflammation, oxidative stress [1], and metabolic abnormalities [2]. Obesity represents a major risk factor to develop OSA [3]–[4]. Sleep apnea is also linked with the risk of incident hypertension [5], metabolic syndrome [6] and with lower insulin sensitivity independent of obesity [7]. A recent study by Pamidi et al. [8] demonstrated that sleep apnea per se is able to decrease insulin sensitivity even in otherwise healthy young men of normal weight.

Continuous positive airway pressure (CPAP), the first line therapy of sleep apnea, reduces the risk of incident cardiovascular events and mortality [5]. CPAP efficacy is still debated regarding metabolic outcomes. It has been recently proposed by Sharma et al. [9], in a cross-over, sham-controlled study, that CPAP treatment in apneic men with the metabolic syndrome could improve metabolic abnormalities and reduce the frequency of metabolic syndrome; however, these results were reported in a highly selected population of drug-naïve patients. In contrast, Hoyos et al. [10] did not found any improvement in insulin sensitivity after three-month of CPAP treatment compared to sham CPAP, although 6-month of CPAP treatment (uncontrolled part of the study) allowed to achieve a significant improvement in insulin sensibility and an increase in lean mass.

Several studies have also shown that waist circumference, waist-on-hip ratio and visceral adiposity were increased in apneic patients compared to control patients of similar body mass index (BMI) [11]–[12]. Increased visceral adiposity leading to ectopic fat depots induces insulin resistance and represents also a well-known cardiometabolic risk factor [13]. Thus, excess visceral fat is likely to participate to the metabolic abnormalities associated with sleep apnea. For instance, Sharma et al. [9], who demonstrated that CPAP therapy was able to partially reverse metabolic disorders in apneic patients, showed that, meanwhile, CPAP treatment was associated with body weight loss and reduction in visceral adiposity. Such a positive effect on body weight and abdominal fat under CPAP treatment was not found by Hoyos et al. [10] and insulin sensitivity did not improved, accordingly. Thus, whereas association between obstructive sleep apnea and metabolic abnormalities has been clearly documented, the respective contribution of obstructive sleep apnea and excess visceral fat to the cardiometabolic risk is not clearly established.

To address this question, the present study recruited normal or overweight, but non obese men with sleep apnea and assessed the respective role of excess abdominal adiposity vs. severity of nocturnal hypoxia on insulin sensitivity and cardiometabolic risk markers.

## Materials and Methods

Thirty-eight adult men without obesity (BMI <30 kg/m^2^), referred for suspicion of sleep apnea, were prospectively included in the study if they were confirmed as suffering from OSA. Men having known hypothyroidism, diabetes (treated or newly diagnosed), treated hypertension or treated dyslipidemia were excluded. Participants performed a 75 g oral glucose tolerance test (OGTT) and biologic measurements to assess lipid, inflammatory and redox plasma profiles. All patients signed an informed consent and the study was approved by institutional ethic committee (Sud-Est V, France).

### Anthropometric Measurements and Body Composition

Height, weight, neck, hip and waist circumferences [14] were measured according to standardized procedures as well as sitting blood pressure. The threshold of 94 cm of waist circumference [15], defining excess abdominal adiposity, was used to define two groups of men: one group of men with OSA and “high-waist circumference” (HWC, waist circumference >94 cm) and one group of men with OSA and “low-waist circumference” (LWC, waist circumference ≤94 cm).

### Polysomnography

An overnight polysomnography was performed in all patients including electroencephalography, bilateral electrooculography, chin and leg electromyography, electrocardiography, airflow by nasal pressure transducer and oronasal thermistor, thoracic and abdominal respiratory efforts, and oxygen saturation (SpO2) by pulse oximetry. Sleep and respiratory events in each patient were recorded according to standard criteria [16]. OSA was defined when apnea-hypopnea index (AHI), i.e., number of apnea plus hypopnea per hour of sleep, was above 15 events/h. The severity of sleep apnea was also evaluated by the average degree of oxyhemoglobin desaturation during the night (mean nocturnal SpO_2_) and by the nighttime spent under 90% of SpO_2_. Sleep stages were visually scored as rapid eye movement (REM), Stage 1 and 2 of non-REM sleep and slow-wave sleep.

### 75g-OGTT

After a 12-hour overnight fast, participants were subjected to a 75 g oral glucose load. Blood samples were taken at 0, 30, 60, 90 and 120 min for the measurement of plasma glucose and insulin concentrations. Plasma glucose was measured enzymatically on Modular® analyser (glucose hexokinase method, Roche Diagnostics, Germany), whereas plasma insulin was determined by immunoradiometric assay (Bis-Insulin IRMA®, CisBio, France) for which detection limit was 0.2 µIU/mL and intra- and inter-reproducibility were 3.8% and 8%, respectively. The OGTT was used to exclude patient with diabetes, defined as fasting glucose ≥7 mmol/L or 120 min OGTT-glucose ≥11.1 mmol/L [17].

The total glucose and insulin areas under the curve (AUC) of OGTT were determined by the trapezoid method between 0 and 120 min. The homeostasis model assessment resistance index (HOMA-IR) [18] was calculated from fasting values of glucose and insulin by the following equation: insulin(µIU/mL)*glucose(mmol/L)/22.5. The insulin sensitivity index of Matsuda (ISI Matsuda) is an OGTT index calculated using formulas adapted from euglycemic hyperinsulinemic clamp studies: 10000/√(glucose at T0*insulin at T0*mean glucose*mean insulin) with plasma glucose in mg/dL and plasma insulin in mIU/L. ISI Matsuda estimates global insulin sensitivity [19]. The AUC *insulin*/AUC *glucose* ratio was calculated to estimate pancreatic insulin secretion in response to plasma glucose level and is presented as the “insulin secretion index”.

### Plasma Lipid Profile

Plasma triglycerides, total cholesterol and HDL-cholesterol were determined using enzymatic colorimetric methods on Modular® analyzer (Roche Diagnostics), on blood sample collected after a 12 h overnight fast. LDL-cholesterol was calculated using the Friedewald formula [Total cholesterol − (HDL-cholesterol+triglycerides/5)]. LDL particle size was determined by polyacrylamide gradient gel electrophoresis.

### Plasma Inflammatory and Redox Profiles

Highly sensitive C-reactive protein (hs-CRP) levels were measured in plasma by immunonephelemetry on BNII® analyser (Dade Behring, USA). Plasma hs-CRP levels >10 mg/L were excluded from the analyses [20]. Plasma homocysteine was measured using liquid chromatography–tandem mass spectrometry (LC–MS/MS). To avoid the in vitro release of homocysteine from red blood cells, all blood samples were immediately centrifuged at 4°C after venipuncture, and aliquots of newly separated plasma were quickly frozen at −80°C until tested. The total plasma antioxidant status (TAS) was measured by a chemiluminescent technique using a Hitachi 904® analyzer (Roche Diagnostics, France) with a Total Antioxidant Status Randox® kit (Randox Laboratories Ltd., Roissy, France). Glutathione peroxidase (GPx; EC 1.11.1.9) activity determination was measured in plasma by spectrophotometry (Hitachi 904®) in the presence of reduced glutathione, glutathione reductase, and NADPH.

### Statistical Analysis

Results are expressed as means (SD) for normally distributed variables and as median (interquartile range) for not normally distributed variables. The normal distribution of residuals was verified by stem and leaf plots and by the Shapiro-Wilk tests. Parameters with skewed distributions (fasting plasma insulin, AUC *insulin*, Insulin secretion index, HOMA-IR, triglycerides, HDL-cholesterol, hs-CRP, TAS) were transformed by log for analyses. Two groups separating participants according to their waist circumference (below or equal to 94 cm vs. above 94 cm) were compared. Comparisons between groups were made by one-way ANOVA. Pearson univariate correlations were computed to address the association between BMI, waist circumference and mean nocturnal SpO_2_ with cardiometabolic risk markers, because these variables were significantly different between the two groups. To address the respective contribution of abdominal adiposity, BMI and mean SpO_2_ during the night to the variance of relevant variables, we built two multivariable linear regression model including waist circumference and mean nocturnal SpO_2_ or BMI and mean nocturnal SpO_2_ as independent variables, and systolic blood pressure, glucose/insulin homeostasis indices, and hs-CRP as dependent variables. Waist circumference and BMI were not added in the same model because of their high co-linearity (r = 0.80). The significance level was set at p<0.05. All analyses were performed using the SAS statistical package version 9.2 (SAS Institute, Cary, NC, USA).

## Results

**Figure 1:**
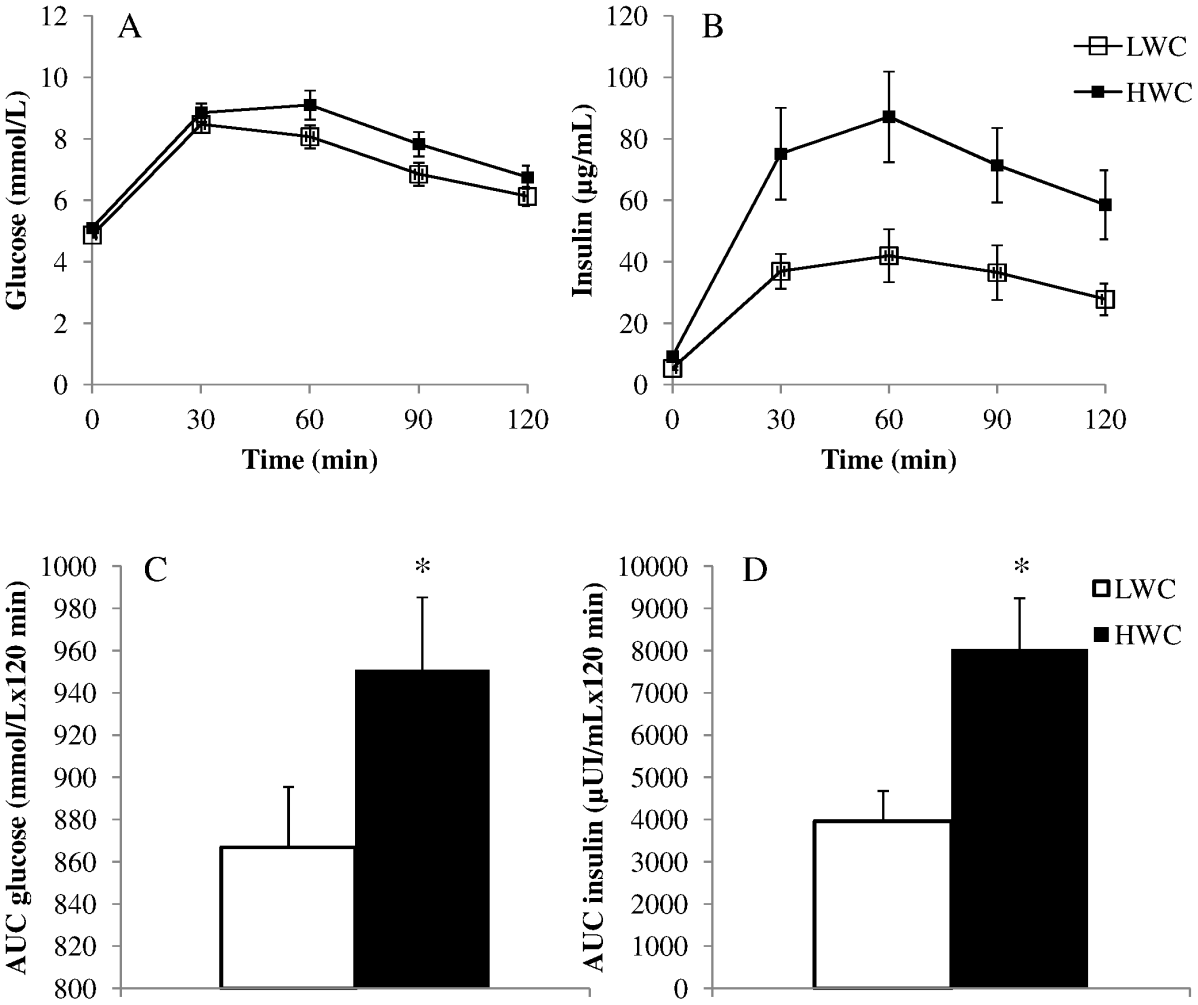
Oral glucose tolerance tests in men with obstructive sleep apnea and high waist circumference or low waist circumference. Plasma glucose (A) and plasma insulin (B) levels in men with obstructive sleep apnea syndrome and high waist circumference (HWC) or low waist circumference (LWC) during 120 minutes of the 75 g oral glucose tolerance test. Area under the curve (AUC) of plasma glucose (C) and plasma insulin (D) are compared between men having obstructive sleep apnea syndrome and HWC vs. LWC. *p<0.05.

Characteristics of all men, those with sleep apnea and HWC (n = 21) and those with sleep apnea and LWC (n = 17) are presented in Table 1. Among these normal or overweight patients with OSA, those with a HWC had higher BMI, neck and hip circumferences, as well as waist/hip ratio than those with LWC. Apneic men with HWC also had a deteriorated cardiometabolic risk profile compared to apneic men with LWC, presenting with higher systolic blood pressure, fasting plasma glucose and insulin, as well as with higher AUC *glucose* and AUC *insulin* (Figure 1). Accordingly, men with OSA and HWC had higher hepatic insulin resistance as reflected by higher HOMA-IR levels, and lower global insulin sensitivity as reflected by lower levels of ISI Matsuda compared to apneic men with LWC. In response to this lower insulin sensitivity, insulin secretion estimated by the insulin secretion index was higher in men with OSA and HWC than in apneic men with LWC.

**Table 1:** Characteristics of patients.

	Total	Apneic men with LWC	Apneic men with HWC	p value
	n = 38	n = 17	n = 21	
Age (years)	49 (11)	48 (9)	50 (12)	0.474
Weight (kg)	83.1 (8.3)	77.5 (7.2)	87.6 (6.2)	**<0.001**
BMI (kg/m^2^)	26.6 (2.7)	24.9 (2.4)	28.0 (2.1)	**<0.001**
Waist circumference (cm)	97.4 (7.8)	89.5 (4.7)	102.7 (4.0)	**<0.001**
Neck circumference (cm)	40.9 (1.9)	39.6 (1.3)	41.8 (1.8)	**<0.001**
Hip circumference (cm)	106.6 (5.8)	101.6 (3.7)	109.9 (4.3)	**<0.001**
Waist on hip ratio	0.91 (0.05)	0.88 (0.04)	0.94 (0.05)	**<0.001**
SBP (mmHg)	134 (14)	129 (15)	138 (12)	**0.044**
DBP (mmHg)	78 (71–84)	75 (9)	80 (11)	0.147
**Smoking status**				0.198
non smoker, n (%)	3 (7.9)	10 (58.8)	6 (28.6)	
former smoker, n (%)	19 (50.0)	6 (35.3)	13 (61.9)	
active smoker, n (%)	16 (42.1)	1 (5.9)	2 (9.5)	
**Plasma glucose/insulin homeostasis**				
Fasting plasma glucose (mmol/l)	4.99 (0.35)	4.87 (0.33)	5.06 (0.34)	**0.047**
Fasting plasma insulin (µUI/ml)	6.31 (4.18–9.94)	4.34 (3.52–6.45)	7.94 (4.94–12.22)	**0.004**
120 min OGTT-glucose (mmol/L)	6.47 (1.56)	6.13 (1.30)	6.74 (1.73)	0.231
AUC *glucose* (mmol/L×120 min)	913 (145)	867 (118)	951 (157)	**0.075**
AUC *insulin* (µUI/mL×120 min)	4381 (2948–7422)	3273 (2713–4225)	6769 (4496–10195)	**0.004**
Insulin secretion index	5.21 (3.21–8.16)	3.92 (2.95–5.15)	6.72 (4.81–10.20)	**0.007**
HOMA-IR	1.47 (0.89–2.29)	0.91 (0.73–1.48)	1.74 (1.09–2.93)	**0.003**
ISI Matsuda	7.21 (4.19)	9.22 (3.86)	5.57 (3.78)	**0.006**
**Plasma lipid profile**				
Total-cholesterol (g/l)	2.18 (0.31)	2.03 (0.33)	2.30 (0.24)	**0.005**
HDL-cholesterol (g/l)	0.55 (0.48–0.67)	0.58 (0.54–068)	0.53 (0.48–0.58)	0.111
LDL-cholesterol (g/l)	1.34 (0.30)	1.26 (0.34)	1.41 (0.26)	0.127
LDL-chol size (Angströms)	270 (4)	271 (3)	269 (4)	0.188
Triglycerides (g/l)	1.09 (0.77–1.41)	0.77 (0.68–1.05)	1.26 (1.12–2.00)	**<0.001**
**Plasma inflammatory and redox profile**				
Hs-CRP (mg/L)	1.40 (0.70–2.80)	0.90 (0.50–1.60)	1.90 (1.10–3.10)	**0.014**
Homocysteine (µmol/L)	11.89 (3.22)	11.07 (3.12)	12.55 (3.22)	0.162
TAS (mmol/L)	1.44 (1.22–1.56)	1.50 (1.43–1.58)	1.35 (1.22–1.45)	0.358
GPx (U/L)	398 (52)	397 (43)	399 (59)	0.891
**Sleep characteristics**				
Epworth score	11 (5)	10 (5)	13 (5)	0.141
Total sleep time (min)	372 (51)	375 (57)	371 (46)	0.841
Stage 1–2 (% du TST)	70.5 (9.1)	70.3 (6.8)	69.3 (11.7)	0.768
Slow wave sleep (% du TST)	4.3 (1.4–9.8)	6.7 (5.6)	6.7 (7.4)	0.982
REM sleep (% du TST)	22.9 (6.3)	22.9 (6.0)	23.4 (7.1)	0.841
AHI total (events/h)	42.4 (17.1)	37.9 (12.8)	46.0 (19.5)	0.151
Mean SpO_2_ (%)	94 (93–95)	94 (94–95)	93 (92–94)	**0.003**
Time spent under 90% of SpO_2_ (%)	1.00 (0.15–4.00)	0.35 (0.00–1.15)	2.70 (0.50–12.60)	**0.028**

Abbreviations: HWC: High waist circumference, LWC: Low waist circumference, BMI: body mass index, SBP: Sleep blood pressure, DBP: diastolic blood pressure, OGTT: oral glucose tolerance test, AUC: area under the curve, ISI: insulin sensitivity index, HOMA-IR: HOMA-resistance index, Hs-CRP: high sensitive CRP, TAS: total antioxidant status, GPx: plasma glutathione peroxidase, REM: rapid eyes movement, AHI: apnea-hypopnea index.

Total-cholesterol, triglycerides levels and hs-CRP were higher in apneic men with HWC whereas 120 min-OGTT glucose, HDL-cholesterol, LDL-cholesterol, homocysteine, TAS and GPx were not different between the two groups.

The subjective daytime sleepiness (Epworth score) and sleep structure were not different between the two groups. The severity of sleep apnea syndrome as measured by AHI was not different according to the presence of excess abdominal adiposity; however, apneic men with HWC presented a lower mean nocturnal SpO_2_ than apneic men with LWC. In addition, considering these normal or overweight apneic men as a whole, waist circumference was found to be negatively correlated to mean nocturnal SpO_2_ (r = −0.43, p = 0.011), but not to AHI. Their BMI, however, was associated neither with mean nocturnal SpO_2_, nor with AHI.

**Figure 2:**
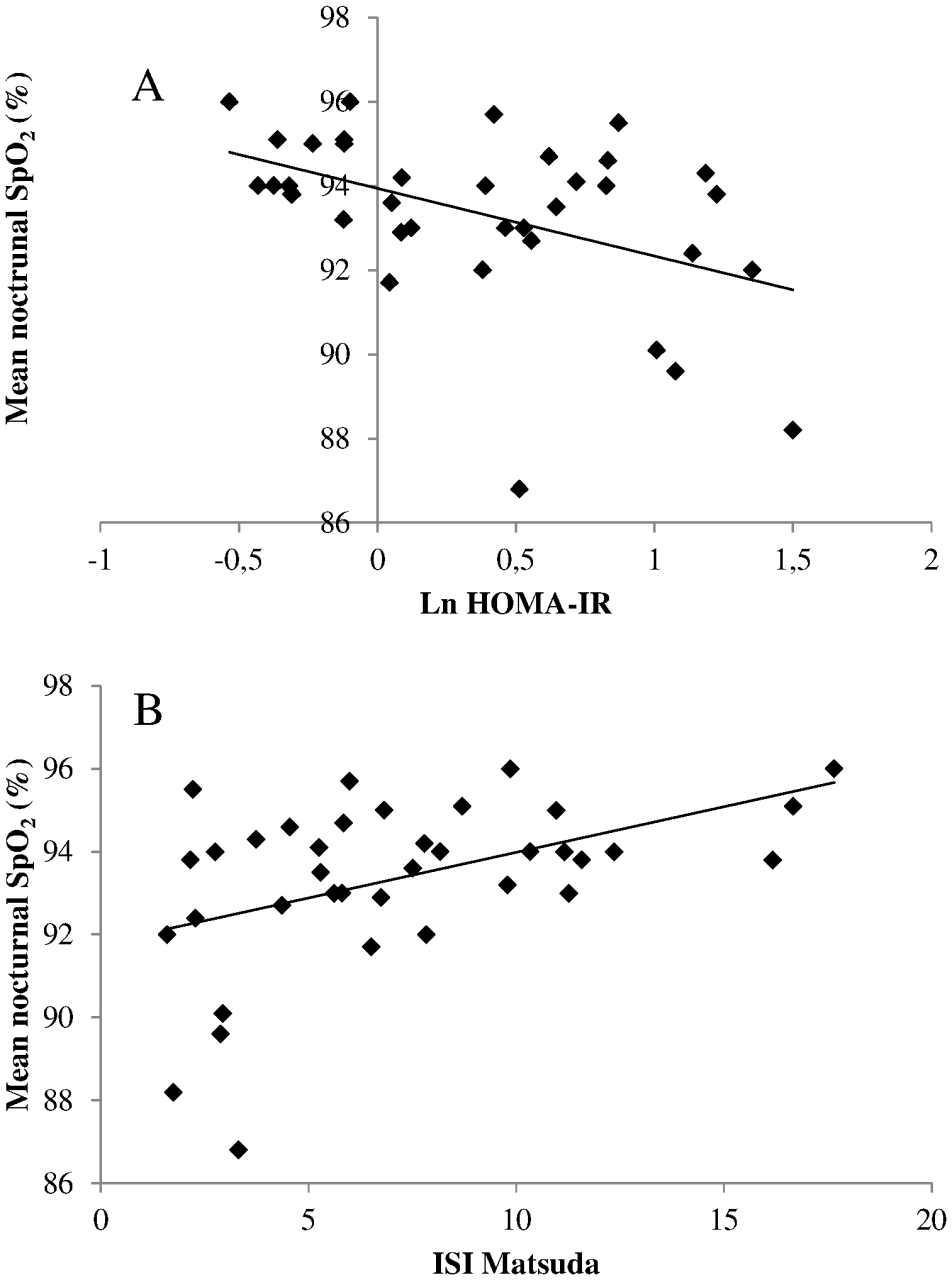
Univariate linear correlation between nocturnal hypoxia and insulin resistance. The average degree of oxyhemoglobin desaturation during the night (mean nocturnal SpO_2_) was negatively correlated to the degree of insulin resistance (A) as estimated by HOMA-IR (r = −0.46, p = 0.004) and positively correlated to the degree of insulin sensitivity (B) as estimated by the insulin sensitivity index of Matsuda (ISI Matsuda, r = 0.46, p = 0.004).

Since apneic men with HWC differed from apneic men with LWC by anthropometric markers of adiposity and by the severity of their sleep breathing disorder-related hypoxia, univariate Pearson correlations were computed to look for specific association between BMI, waist circumference and mean nocturnal SpO_2_ with cardiometabolic risk markers (Table 2 and Figure 2). Systolic blood pressure, plasma glucose/insulin homeostasis indices and hs-CRP were correlated with mean nocturnal SpO_2_ and/or with BMI and waist circumference. Diastolic blood pressure, plasma lipid or redox profiles were not associated with these markers of adiposity/sleep breathing disorder-related hypoxia.

**Table 2:** Univariate Pearson correlations.

	BMI	Waist circumference	Mean nocturnal SpO_2_
Systolic blood pressure	0.35^a^	0.39^a^	
Diastolic blood pressure			
**Plasma glucose/insulin homeostasis**			
Fasting plasma glucose	0.36^a^	0.37^a^	−0.54^a^
Ln Fasting plasma insulin	0.39^a^	0.45^a^	−0.41^a^
120 min OGTT-glucose	0.32^a^	0.37^a^	
AUC *glucose*		0.45^a^	
Ln AUC *insulin*		0.37^a^	−0.49^a^
**Ln Insulin secretion index**			−0.47^a^
Ln HOMA-IR	0.42^a^	0.47^a^	−0.46^a^
ISI Matsuda	−0.36^a^	−0.43^a^	0.46^a^
Plasma lipid profile			
Total-cholesterol			
Ln HDL-cholesterol			
LDL-cholesterol			
LDL-chol size			
Ln Triglycerides			
**Plasma inflammatory and redox profiles**			
Ln Hs-CRP	0.39^a^	0.46^a^	
Homocysteine			
TAS			
GPx			

Abbreviations: OGTT: oral glucose tolerance test, AUC: area under the curve, ISI: insulin sensitivity index, HOMA-IR: HOMA-resistance index, Hs-CRP: high sensitive CRP, TAS: total antioxidant status, GPx: plasma glutathione peroxidase. p<0.05 are reported by a in exponent.

To assess the relative contribution of markers of adiposity and mean nocturnal SpO_2_ to systolic blood pressure, plasma glucose/insulin homeostasis indices and hs-CRP, we built two multivariable regression models, with waist circumference and mean nocturnal SpO_2_ or BMI and mean nocturnal SpO_2_ as independent variables (Table 3 and 4, respectively). Systolic blood pressure, fasting plasma insulin and 120 min-OGTT glucose were independently associated neither with mean nocturnal SpO_2_, nor with waist circumference. However, fasting plasma glucose, AUC *insulin*, HOMA-IR, ISI Matsuda and the insulin secretion index were independently associated with the mean nocturnal SpO_2_, but not with waist circumference. AUC *glucose* and hs-CRP were independently associated with waist circumference, but not with mean nocturnal SpO_2_ (Table 3).

**Table 3:** Multivariable linear regression analyses: relative contribution of mean nocturnal SpO_2_ and waist circumference.

Dependent variables	Independent variables	β coefficient	95 CI		p valeur	AIR	R^2^ (×100%)
Systolic blood pressure	Mean SpO_2_	−0.58334	−3.10297	1.93628	0.640	178	10
	WC	0.61021	−0.04728	1.26769	0.068		
Fasting plasma glucose	Mean SpO_2_	−0.09558	−0.15068	–0.04048	**0.001**	−82	34
	WC	0.00574	−0.00864	0.02012	0.422		
Ln Fasting plasma insulin	Mean SpO_2_	−0.08048	−0.17479	0.01383	0.092	−45	23
	WC	0.02302	−0.00159	0.04763	0.066		
120 min OGTT-glucose	Mean SpO_2_	−0.08220	−0.37687	0.21246	0.573	32	9
	WC	0.06616	−0.01073	0.14305	0.089		
AUC *glucose*	Mean SpO_2_	−9.30714	−35.3518	16.7375	0.472	337	16
	WC	7.36902	0.57278	14.1652	**0.035**		
Ln AUC *insulin*	Mean SpO_2_	−0.14871	−0.26871	–0.02870	**0.017**	−29	23
	WC	0.01629	−0.01502	0.04761	0.297		
Ln Insulin secretion index	Mean SpO_2_	−0.14017	−0.24940	–0.03093	**0.014**	−35	20
	WC	0.00826	−0.02025	0.03676	0.559		
ISI Matsuda	Mean SpO_2_	0.80507	0.08445	1.52568	**0.030**	93	26
	WC	−0.15181	−0.33986	0.03623	0.110		
Ln HOMA-IR	Mean SpO_2_	−0.09917	−0.19469	–0.00364	**0.042**	−44	28
	WC	0.02419	−0.00074	0.04911	0.057		
Ln Hs-CRP	Mean SpO_2_	−0.00282	−0.16049	0.15485	0.971	−10	16
	WC	0.05187	0.01073	0.09301	**0.015**		

Abbreviations: SBP: Sleep blood pressure, OGTT: oral glucose tolerance test, AUC: area under the curve, ISI: insulin sensitivity index, HOMA-IR: HOMA-resistance index, Hs-CRP: high sensitive CRP, WC: waist circumference.

**Table 4:** Multivariable linear regression analyses: relative contribution of mean nocturnal SpO_2_ and BMI.

Dependent variables	Independent variables	β coefficient	95 CI		p valeur	AIR	R^2^ (x100%)
Systolic Blood pressure	Mean SpO_2_	−0.92439	−3.21200	1.36322	0.417	194	9
	BMI	1.63263	−0.06783	3.33309	0.059		
Fasting plasma glucose	Mean SpO_2_	−0.08430	−0.13502–	0.03359	**0.002**	−88	31
	BMI	0.03150	−0.00619	0.06920	0.099		
Ln Fasting plasma insulin	Mean SpO_2_	−0.09155	−0.17481–	0.00829	**0.032**	−51	22
	BMI	0.06259	0.00071	0.12448	**0.048**		
120 min OGTT-glucose	Mean SpO_2_	−0.13839	−0.40038	0.12361	0.291	34	8
	BMI	0.16365	−0.03110	0.35840	0.097		
AUC *glucose*	Mean SpO_2_	−18.89967	−43.1792	5.37985	0.123	369	8
	BMI	10.73885	−7.30892	28.7866	0.235		
Ln AUC *insulin*	Mean SpO_2_	−0.15442	−0.26094–	0.04790	**0.006**	−33	23
	BMI	0.04673	−0.03245	0.12592	0.239		
Ln Insulin secretion index	Mean SpO_2_	−0.13584	−0.23454–	0.03713	**0.008**	112	19
	BMI	0.03408	−0.03929	0.10745	0.352		
ISI Matsuda	Mean SpO_2_	0.84748	0.20754	1.48743	**0.011**	100	24
	BMI	−0.41225	−0.88794	0.06345	0.087		
Ln HOMA RI	Mean SpO_2_	−0.10795	−0.19263–	0.02328	**0.014**	−50	27
	BMI	0.06907	0.00613	0.13202	**0.032**		
Ln Hs-CRP	Mean SpO_2_	−0.04648	−0.18897	0.09601	0.512	−11	10
	BMI	0.11328	0.00736	0.21920	**0.037**		

Abbreviations: SBP: Sleep blood pressure, OGTT: oral glucose tolerance test, AUC: area under the curve, ISI: insulin sensitivity index, HOMA-IR: HOMA-resistance index, Hs-CRP: high sensitive CRP, BMI: body mass index.

Systolic blood pressure, 120 min-OGTT glucose and AUC *glucose* were independently associated neither with mean nocturnal SpO_2_, nor with BMI. However, fasting plasma glucose, AUC *insulin*, ISI Matsuda and the insulin secretion index were independently associated with the mean nocturnal SpO_2_, but not with BMI. Hs-CRP was independently associated with BMI, but not with mean nocturnal SpO_2._ Fasting insulin and HOMA-IR were independently associated with both BMI and the mean nocturnal SpO_2_ (Table 4).

## Discussion

The non-obese men with OSA and HWC were characterized by a deteriorated cardiometabolic risk profile compared to non-obese men with OSA and LWC. They also presented with a lower mean nocturnal SpO_2_, while severity of their sleep apnea syndrome measured by AHI was not different. Waist circumference and mean nocturnal SpO_2_ were inversely correlated and were both associated with plasma glucose/insulin homeostasis indices: the higher the waist circumference, the lower the mean nocturnal SpO_2_, the lower the insulin sensitivity. Finally, in multivariable regression models, mean nocturnal SpO_2_ and not waist circumference was associated with insulin resistance, as measured by HOMA-IR and ISI Matsuda.

### Excess Visceral Adiposity and Sleep Apnea Syndrome

Approximately half of these non obese apneic patients presented with abdominal obesity. They happen to have more severe nocturnal hypoxia than apneic lean men, but a similar AHI. Several studies have addressed the question whether a specific fat distribution was particularly associated with sleep apnea syndrome, beyond obesity itself. Shinohara et al. [21] demonstrated in 37 obese men and women that visceral adiposity, measured by computed tomography, was correlated to the severity of AHI, independent of total adipose tissue. Vgontzas et al. [22] compared 14 obese men with sleep apnea syndrome with 10 BMI-matched obese men without sleep apnea and found that visceral adiposity was higher in obese men with sleep apnea. Visceral fat accumulation was correlated with mean nocturnal SpO_2_, as we found in the present study, but also with AHI. Schäfer at al. [23] evaluated fat distribution by RMN measures in 60 men attempting sleep clinic for a suspected obstructive sleep apnea. These men were either apneic (defined as AHI>10 events/h) or non apneic, with a BMI ranging from normal weight to grade 3 obesity. Authors found that AHI was significantly correlated with BMI (r = 0.44), intra-abdominal fat (r = 0.29), but neither with subcutaneous fat in the neck region, nor with parapharyngeal fat; however, AHI was not associated with intra-abdominal fat independent of BMI. Association of fat distribution with nocturnal SpO_2_ was not examined. By contrast with these previous studies, the present work excluded obese patients to discriminate abdominal obesity from global obesity. Abdominal adiposity raises the diaphragm and reduces lung volume because lower lung becomes atelectic [24]. This could explain why for a similar AHI the severity of nocturnal hypoxia was increased in men with high waist circumference.

In an observational study, Chin et al. [25] found that six-months of CPAP treatment in patients with OSA was associated with a reduction in visceral fat independent of body weight loss. While the study was not designed on this purpose, Sharma et al. [9] found that 3-months of CPAP compared to Sham-CPAP, in a cross-over randomized clinical trial, was associated with a significant loss of visceral fat, in parallel of a significant body weight loss. On the contrary, Hoyos et al. [10] conducted a three-month controlled study to address the effect of CPAP treatment on visceral fat accumulation and did not found that CPAP treatment induced any change in visceral fat accumulation compared to Sham-CPAP. Finally, Borel et al. [26] have reported that patient having abdominal obesity had less benefits of a lifestyle intervention program aiming to lose weight when they presented with untreated sleep breathing disorders. Thus, previous studies as well as results of the present work suggest that visceral adiposity and sleep apnea syndrome are specifically associated and might interact as a vicious circle; however, whether CPAP treatment could reduce visceral fat by itself is still debated and whether CPAP treatment during lifestyle intervention could improve the results of such programs remains to be evaluated.

The mechanistic links explaining the deleterious interaction between visceral fat and sleep apnea could be related to changes in adipokines and incretins under intermittent hypoxia. In obese mice, local hypoxia at the fat tissue level appears to dysregulate adipokines expression [27]–[28]. In humans, Pasarica et al. [29] measured direct oxygen partial pressure in subcutaneous fat and demonstrated that local hypoxia was strongly associated with adipose tissue inflammation and increased macrophage accumulation. Sleep apnea, as well as reduction in sleep duration is associated with higher levels of ghrelin [30], an orexigenic incretin, that may participate to excess body weight in patients with poor sleep quality. Thus, intermittent hypoxia appears to deteriorate the adipokine profile of adipose tissue, and to increase orexigenic signals. In turn, excess visceral adiposity decreases chest wall compliance and lung volume that favors obstructive sleep apnea [31].

### Excess Visceral Adiposity and Insulin Resistance

Visceral fat is characterized by a high lipolytic rate. Thus, excess visceral adiposity increases delivery of deleterious levels of FFA to the liver via the portal vein leading to elevated hepatic triglyceride concentration and hepatic insulin resistance. Such ectopic fat deposition also occurs in muscle and heart leading to insulin resistance [13]. Thus, visceral adiposity is a stronger correlate of insulin resistance than subcutaneous adiposity [32] and the improvements in insulin sensitivity after lifestyle interventions are mainly driven by the reduction in visceral adiposity [33]. Results of the present study confirm that waist circumference is positively correlated with insulin resistance and other features of the cardiometabolic risk in these non-obese men with obstructive apnea syndrome, as in general population [34].

### Sleep Apnea Syndrome and Insulin Resistance

In addition to the association between abdominal adiposity and decreased insulin sensitivity, we found that mean nocturnal SpO_2_, i.e. nocturnal hypoxia, was inversely correlated with insulin resistance in these non-obese men with obstructive apnea syndrome. Multivariable analyses showed that nocturnal hypoxia and not abdominal adiposity was independently associated with hepatic insulin resistance (estimated by HOMA-IR) and global insulin sensitivity (estimated by ISI Matsuda). Several studies sustain the hypothesis that sleep apnea per se deteriorates insulin sensitivity, independent of obesity [35]. Punjabi et al. [36] have measured insulin sensitivity by the frequently sampled intravenous glucose tolerance test in 118 patients undergoing polysomnography and found that insulin sensitivity measured by the sensitivity index (S_I_) was associated with the severity of AHI and nocturnal hypoxia, independent of percentage of fat mass. Ip et al. [7] found that markers of sleep apnea severity as AHI and nocturnal minimum SpO_2_ were associated with insulin resistance, estimated by fasting insulin and HOMA-IR, in 270 patients undergoing polysomnography. In multivariable analyses, both marker of sleep apnea severity and BMI/waist circumference were associated with fasting insulin and HOMA-IR. In the present work, despite univariate association of both nocturnal hypoxia and abdominal obesity with markers of insulin resistance, only nocturnal hypoxia remained independently associated with insulin resistance in multivariable analyses. Compared to the study by Ip et al. in which patients had a large range of BMI, from normal weight to obesity, the present work selected patients of normal weight or overweight but not obese. That might be the reason why association between sleep apnea marker of severity and insulin resistance appeared stronger than the association between abdominal obesity and insulin resistance. Indeed, a recent study by Pamidi et al. [8] looked for association of sleep breathing disorders and insulin resistance in healthy young men of normal weight. Despite these men presented with only mild sleep apnea (AHI between 5 to 15 events/hour), they had a lower insulin sensitivity measured by ISI Matsuda index than non apneic controls. Authors found that AHI was inversely correlated with ISI Matsuda. However, in these young men of normal weight, no imagery or anthropometric measure was performed to look for a potential difference in visceral fat accumulation between apneic and non apneic men.

Thus, results of the present study suggest that insulin sensitivity is deteriorated in proportion of the severity of nocturnal hypoxia. In abdominally obese men, the severity of sleep apnea seems associated with additional deleterious effect on insulin sensitivity beyond insulin resistance related to abdominal adiposity. The present results suggest that in non obese patients with OSA and excess abdominal fat, nocturnal hypoxia but not AHI may drive the deterioration of insulin sensitivity.

### Study Limitations

Our sample size was relatively small due to highly selective eligibility criteria, comparable to the number of patients included in the study by Pamidi et al. in non-obese apneic patients [8]. We used indirect, but validated, OGTT-based measures of insulin sensitivity and secretion. Patients with known hypothyroidism were excluded but biological hypothyroidism was not systematically assessed. Thus, some overlapping between hypothyroidism-related sleep breathing disorders and primary OSA could have happen. Abdominal obesity was estimated by anthropometric measurements without imagery techniques that would have allowed differentiating between subcutaneous abdominal fat and visceral fat; however, waist circumference is a validated correlate of visceral fat and a recognized marker of cardiometabolic risk [34]. Waist circumference is more likely to be used by clinician allowing better exporting the present results to routine clinical practice. The present study was designed to specifically address the respective association of abdominal adiposity and severity markers of sleep apnea with plasma glucose/insulin homeostasis indices. Thus, the study focused on normal weight and overweight patients in which waist circumference is a good correlate to differentiate “healthy” and “deleterious” fat distribution, whereas this marker is less relevant in obese patient. In addition, selection criteria to diagnose sleep apnea was set at 15 events/hour, defining mild to severe apnea that qualifies patients to benefit of CPAP treatment, whereas lots of previous studies have also considered moderate apnea (above 5 events/hour) which is a threshold dissociated from clinical practice.

### Conclusions

Non obese patients are likely to be under-diagnosed for sleep breathing disorders because sleep apnea syndrome is a well-known co-morbidity of obesity. However, the present results showed that the severity of sleep apnea in these normal weight and overweight patients was correlated with insulin resistance, increasing the risk to further develop type 2 diabetes and cardiovascular complications. Men presenting with abdominal obesity had a deteriorated cardiometabolic risk profile compared to lean men as well as a more severe nocturnal hypoxia that seemed to drive the deleterious effect on insulin sensitivity.
